# Pancreatic Neuroendocrine Tumor with Benign Serous Cystadenoma: A Rare Entity

**DOI:** 10.1155/2021/9979998

**Published:** 2021-08-04

**Authors:** Randhir Sagar Yadav, Ashik Pokharel, Shumneva Shrestha, Ashbita Pokharel, Deepshikha Gaire, Sumita Pradhan, Prasan Bir Singh Kansakar

**Affiliations:** ^1^Maharajgunj Medical Campus, Institute of Medicine, Tribhuvan University, Kathmandu, Nepal; ^2^Department of Gastrointestinal and General Surgery, Tribhuvan University Teaching Hospital, Kathmandu, Nepal; ^3^Department of Pathology, Tribhuvan University Teaching Hospital, Kathmandu, Nepal

## Abstract

Mixed serous-neuroendocrine neoplasm constitutes pancreatic serous cystic neoplasms and pancreatic neuroendocrine tumor, two tumor components with different underlying pathologies. The differentiation of these tumors is important as the management and prognosis depend on the pancreatic neuroendocrine tumor component. We report a case of mixed serous-neuroendocrine neoplasm in a 47-year-old female who presented with epigastric pain abdomen for two years. Imaging studies, tumor markers, thorough systemic evaluation, surgical resection, histopathological examination, and timely follow-up constituted our management approach. A 4 cm × 4 cm mass in the distal pancreas with multiple cysts in the pancreatic parenchyma containing serous fluid on distal pancreatectomy and splenectomy was found. The histopathological examination revealed combined benign serous cystadenoma and neuroendocrine tumor. She did not have any recurrence or metastasis by four years of follow-up.

## 1. Introduction

Pancreatic cystic lesions are relatively rare, which are mostly serous and mucinous subtypes [1]. Pancreatic serous cystic neoplasms (PSCNs) are a benign yet very rare form of neoplasms that account for 1-2% of all pancreatic tumors [2, 3]. PSCNs were further classified into five subtypes, namely, serous microcystic cystadenoma, serous oligocystic cystadenoma, solid serous adenoma, Von Hippel-Lindau (VHL) syndrome-associated serous cystic neoplasm, and mixed serous-neuroendocrine neoplasm (MSNN) by the World Health Organization Digestive system tumor classification. MSNN constitutes of the tumor with two components of different pathologies which are PSCN and pancreatic neuroendocrine tumor (PanNET) [4].

## 2. Case Report

**Figure 1:**
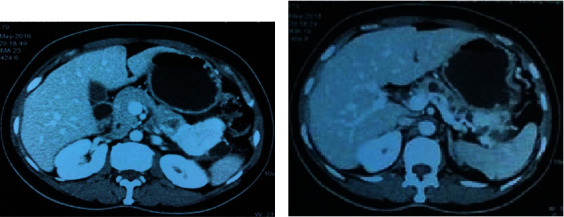
(a, b) Contrast-enhanced computed tomography (CECT) of abdomen and pelvis showed intensely enhancing mass in the tail of pancreas along with multiple cysts with calcific foci in the body of the pancreas suggestive of a neuroendocrine tumor with multiple cysts in the pancreas.

**Figure 2:**
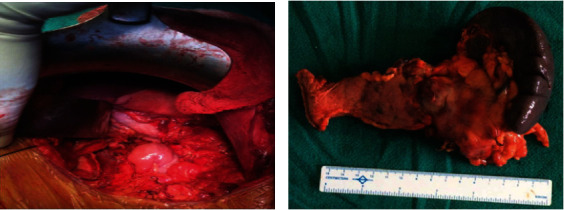
(a, b) The operative finding showing a 4 cm × 4 cm mass in the distal pancreas with multiple cysts in the pancreatic parenchyma containing serous fluid and specimen following distal pancreatectomy and splenectomy.

**Figure 3:**
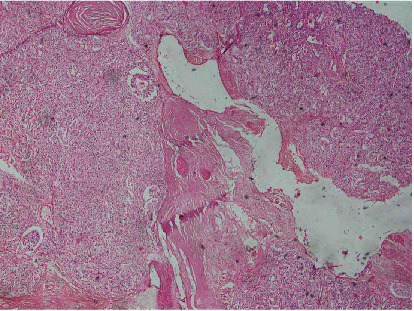
Image showing serous cystic neoplasm composed of cystic structure focally lined by cuboidal cells with bland nuclear features and clear cytoplasm (H and E, ×200).

**Figure 4:**
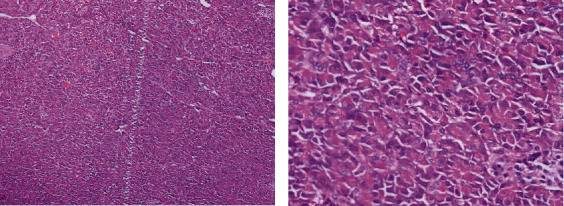
(a, b) Image showing neuroendocrine neoplasm, tumor cells arranged in insular, ribbons, and cords. No atypia and pleomorphism noted. Mitosis is scant (H and E, 40x). Tumor cells with mild nuclear atypia, moderate amount of eosinophilic granular cytoplasm (B, H, and E, 400x).

A 47-year-old female presented with pain abdomen especially in the epigastrium for two years. She denied any history of vomiting, jaundice, anorexia, or weight loss. She had a history of type 2 diabetes mellitus for five years controlled under oral hypoglycemic drugs. Physical examination did not reveal any abnormal findings. Laboratory evaluation showed a normal level of complete blood count, comprehensive metabolic panel, and amylase. Furthermore, tumor markers CEA (1.46 ng/ml; normal <2.5 ng/ml), CA 19-9 (14.6 IU/ml; normal <37 IU/ml), and CA 125 (30.8 IU/ml; normal <46 IU/ml) were normal, while Chromogranin A was elevated (172 ng/ml; normal <100 ng/ml). The contrast-enhanced computed tomography (CECT) of the abdomen and pelvis showed an intensely enhancing mass in the tail of the pancreas along with multiple cysts with calcific foci in the body of the pancreas suggestive of a neuroendocrine tumor with multiple cysts in the pancreas (Figures 1(a) and 1(b)). Based on the evaluation, with a diagnosis of cystic pancreatic neoplasm, our patient underwent distal pancreatectomy and splenectomy. The operative finding showed a 4 cm × 4 cm mass in the distal pancreas with multiple cysts in the pancreatic parenchyma containing serous fluid (Figures 2(a) and 2(b)). The postoperative period was uneventful except grade A postoperative pancreatic fistula. The patient was discharged on the seventh postoperative day. The histopathological examination revealed combined benign serous cystadenoma (Figure 3) and neuroendocrine tumor, grade 1 (Figure 4). Cyst was lined by flattened to cuboidal cells with moderate amount of clear cytoplasm. Neuroendocrine components showed tumor cells arranged in lobule, trabecular, and rosette patterns. Cells showed central nucleus with finely stippled chromatin and moderate amount of eosinophilic granular cytoplasm. Atypia, pleomorphism, and mitosis was not noted. Mitosis constituted 0-1/10 HPF with Ki-67 index of 1%. The resection margins were free of tumor, and all lymph nodes were tumor-free (0/5). Therefore, a diagnosis of MSNN was established. Throughout the four years of regular follow-up, her pain abdomen did not recur, neither was any evidence of tumor recurrence or metastasis.

## 3. Discussion

Despite its rarity, PSCNs are occasionally found with other concurrent tumors like PanNET, pancreatic ductal adenocarcinoma, intraductal papillary mucinous neoplasm, and metastatic tumors [5]. Among these PanNET, the most common type found mixed tumor with PSCNs is called MSNN [4]. Reid et al. found a concurrence of 6% of PanNET [6] in a large cohort of PSCNs (193) [5].

In the case report and literature review of 15 cases, Li et al. categorized MSNN into four subtypes, namely, (1) diffuse subtype where numerous PSCNs occupied the entire pancreas along with one or more PanNETs, located in any part of the pancreas; (2) mixed subtype having two different tumors combined in a mass which cannot be distinctly separated; (3) solitary subtype having different components coinciding in the pancreas without any intermingling area; and (4) collision subtype with two different lesions separated from each other in most areas, but having a partially intermixed or overlapping zone [7]. Further, Xu et al. published a case report with a review of 22 cases including 15 cases reviewed by Li et al. Among the 22 cases, diffuse, multiple, and isolated PSCNs were seen in nine (41%), two (9%), and 11 (50%), cases, respectively. Likewise, based on the subtypes categorized by Li et al., most patients had diffuse subtype (41%; 9/22) followed by mixed (27%; 6/22), solitary (23%; 5/22), and collision subtypes (9%; 2/22). Among these, there was only one case, each of mixed and collision subtype in the pancreatic tail. All cases had solitary PanNET [8]. In a recent classification in 2019, WHO had classified serous cystadenoma as microcystic, macrocystic (oligocystic), solid, and diffuse based on gross features. Microcystic are usually well circumscribed with numerous tiny cysts and a central scar. Oligocystic type usually composed of 1 to 10 cysts and lacks a central scar. Solid types are well circumscribed with complete absence of cyst formation. Diffuse types completely replace the pancreas with cysts and are usually associated with VHL. Similarly, the histological types of serous cystadenoma are classified as microcystic, macrocystic, solid, VHL associated, and mixed serous neuroendocrine neoplasm [9].

These MSNN cases showed a higher incidence in females, and it mostly presented with pain abdomen, nausea/vomiting, weight loss, and jaundice [7, 8]. The laboratory investigations were found normal [7]. Our patient was also a female who presented with pain abdomen while routine laboratory investigations were also within the normal range.

Transabdominal ultrasonography is the first imaging study done for a pancreatic lesion. Multidetector computed tomography (MDCT) is the gold standard test for detecting solid pancreatic tumors. Pancreatic cystic lesions are better visualized on magnetic resonance imaging (MRI), while MR with cholangiopancreatography (MRCP) is the gold standard investigation to identify the cystic pancreatic lesion [10]. Similarly, state-of-art imaging plays a vital role in the detection, diagnosis, and staging of PanNET [11]. The abovementioned reported cases of MSNN had undergone ultrasonography, enhanced thin-sliced spiral CT, and magnetic resonance imaging scans which were suggestive of pancreatic mass [7]. Even though histopathology can easily differentiate PSCN and PanNET [5], their differential diagnoses can be challenging if the biopsy samples are small [8]. Also, PSCNs can be misdiagnosed as PanNET if the PSCN is a solid variant or serous adenoma [5]. So the presence of PanNET should be carefully examined in a specimen of PSCN [8]. Moreover, tumor markers also help differentiate PSCN and PanNET. PSCN does not show reactivity to Chromogranin A [12], while it shows a positive result on epithelial markers CK19 and CK7 and possibly other markers (*α*-inhibin and calponin) [6, 13]. PSCNs should be adequately distinguished from mucinous cysts which are malignant [7]. In our case, CECT was suggestive of a neuroendocrine tumor with multiple cysts in the pancreas. Chromogranin A was high, suggestive of a neuroendocrine tumor particularly in an absence of other causes that could elevate Chromogranin A [14] in our patient. Additionally, other tumor markers like CEA and CA19-9 were found normal. Finally, the diagnosis was confirmed on histopathological examination which revealed the benign form of tumors.

PSCN in itself is benign [2] and the PSCN component of MSNN is benign too [8]. But the PanNET is a low-grade malignancy [4]. Moreover, MSNN has a higher malignant potential than PSCNs and PanNET alone [7, 15]. Therefore, it is imperative to differentiate PSCN from PanNET as the treatment and prognosis of MSNN depend on the presence of PanNET. PSCN patients are kept under observation [2], whereas surgery is the first-line treatment for the resectable PanNET [7, 16]. Further, among PanNET less than 2 cm, the choice of surgical or conservative management is debatable [17]. Case review also showed that the MSNN patients underwent radical surgeries like pancreaticoduodenectomy, distal pancreatectomy with splenectomy, and total pancreatectomy [7]. Our patient also underwent distal pancreatectomy with splenectomy.

MSNN with associated VHL associated syndrome requires further consideration as VHL syndrome-associated MSNN presents at a younger age, has diffuse-type PCSN, has a larger PanNET size, and has other associated tumors (such as renal clear cell carcinoma, cerebellar/retina hemangioblastoma, liver angioma, adrenal pheochromocytoma, and paravertebral paraganglioma) [8]. Moreover, VHL syndrome associated with PanNET has a higher potential for malignancy [18]. Thus, MSNN patients should be meticulously evaluated for the presence of other lesions and VHL syndrome. On workup, our patient was not found to have any other associated condition. Our patient was followed up according to the standard guidelines [17, 19] and did not show disease progression.

## 4. Conclusion

MSNN constitutes of histopathologically two distinct lesions where thorough evaluation is needed to explore other associated conditions to timely detect and manage. Since the PanNET component of MSNN is a potentially malignant tumor, close follow-up and surveillance are imperative for detecting any recurrence or metastasis.

## Data Availability

All data are within the article.
